# Investigation of inerter-based suspension systems for heavy vehicles

**DOI:** 10.1371/journal.pone.0280290

**Published:** 2023-01-20

**Authors:** Ming Foong Soong, Rahizar Ramli, Ahmad Abdullah Saifizul, Kah Yin Goh, Su Xian Long

**Affiliations:** Faculty of Engineering, Department of Mechanical Engineering, Universiti Malaya, Kuala Lumpur, Malaysia; National University of Sciences and Technology, PAKISTAN

## Abstract

The inerter is a two-terminal component that can be added to the spring-and-damper configuration of a suspension system. It has the property that the force exerted is proportional to the relative acceleration at its terminals. Studies have demonstrated the inerter’s benefit of providing superior vibration isolation when it is used in the vehicle suspension of passenger cars. However, similar benefit on another common vehicle class on the roads, namely heavy vehicles, remain to be shown, as these vehicles have vastly different parameter values than passenger cars. This study is an investigation on the performance improvement brought by an inerter in the suspension of common heavy vehicles. In the study, the parameter values of a truck and a bus were adopted in the quarter vehicle model with two different spring-damper-inerter configurations (parallel and serial inerter), and the improvements in vibration isolation and road holding capability were determined by optimization of inertance. Results show that the inerter is similarly effective in providing the said improvements when implemented on heavy vehicles instead of on passenger cars, judging from reductions in sprung mass acceleration and dynamic tire load. It is also observed that the performance benefit is associated with larger optimum inertance than that for passenger cars. Overall, the inerter has been shown to be beneficial in the parallel and serial configurations, both of which are common and can be practically implemented in the suspension of heavy vehicles.

## Introduction

The suspension system of a vehicle is a system of springs, dampers and linkages which connect between the wheel and the vehicle body. When designed and tuned accordingly, a vehicle suspension can serve the purposes of isolating the vehicle body from vibrations coming from the ground due to road irregularities, as well as maintaining consistent contact between the wheel and the road surface by minimizing the tire’s normal load variations [1]. The former is important for ride comfort (passenger-carrying vehicles) or protection of goods (goods-carrying vehicles), while the latter is important for tire’s road holding ability which indirectly relates to vehicle handling and safety. Given the extensive usage of road vehicles in the transportation scene, a well-performing vehicle suspension is especially important.

Regardless of the complexity of a modern vehicle suspension system, it can currently be generalized to having two major elements: the spring element and the damper element. The former supports the static load of a vehicle and temporarily stores the undesirable energy due to ground vibrations, while the latter dissipates this unwanted energy and literally dampen the vehicle response. Presently, the design and tuning of a vehicle suspension is largely around these two components. However, less known is that apart from spring and damper, there is another two-terminal element known as the inerter [2] that can also join the line-up as a suspension component. Fundamentally, the inerter is a two-terminal, mass-like inertial device that can provide translational inertia by utilizing the rotational inertia of a flywheel and converting it back to translational effect via motion conversion mechanisms, thereby responding to the relative motion of the two terminals. With the addition of inerter, this forms the trio of suspension elements: the spring reacts to relative displacement, the damper reacts to relative velocity, while the inerter reacts to relative acceleration. More importantly, the addition of inerter in a suspension stretches the conventional limit of suspension design and tuning, because of the wider possibility a new element offers, for instance: the various suspension layouts with inerter and the optimization of inertance which is the defining property of the inerter.

The inerter has been shown to be applicable in various areas, some of which include train suspension [3–5], building suspension [6–9], and vehicle suspension [10–16]. In all these applications, benefits to vibration isolation have been reported. For example, in train suspension application, early studies showed that performance improvements and lateral stability could be obtained by employing the inerter [3,4], while the focus was later shifted towards using inerter-based suspension to improve both passenger comfort and track wear [5]. Meanwhile, in building application, the inerter was initially studied as a suspension to building which demonstrated effective vibration suppression [6–8]. The implementation eventually evolved to the tuned mass damper with inerter system as a better alternative to conventional tuned mass damper [9]. In the context of vehicle suspension, it has been shown that the ride and road holding performance can be enhanced by the addition of inerter to the original spring-and-damper setup [10–16]. These studies demonstrate the promising incorporation of inerter in many vehicle suspensions. However, one thing in common is that these concentrated solely on passenger cars, judging from the use of vehicle parameter values that are typical to this class of vehicles. What this means is that the other major category of vehicles, commonly termed as heavy vehicles, have not been explored before with inerter. In general, heavy vehicles consist of several classes of goods-carrying or passenger-carrying vehicles, such as heavy trucks, single-unit-trucks, buses, etc. Although the suspension’s purposes of achieving vibration isolation and road holding ability remain the same, heavy vehicles can have different suspension requirements due to vastly different sprung and unsprung masses and the corresponding different mass ratios. In the context of implementation of inerter which is still new to heavy vehicles, these include suitable suspension layouts with inerter, the optimum inertance values, and the corresponding ride and road holding performance brought by the addition of an inerter.

This study investigates the improvements in vibration isolation (ride) and road holding ability brought by the incorporation of an inerter, specifically concerning heavy vehicles. In the study, the suspension performance criteria were evaluated theoretically, considering two heavy vehicle classes (trucks and buses) as well as common suspension layouts. Through the analysis presented in the sections that follow, it was determined that the inerter, when incorporated in heavy vehicles, is similarly effective in achieving superior vibration isolation. This is important as it translates to better passenger comfort (for buses) and prevention of goods damage (for heavy trucks) in the transportation scene.

## Inerter in vehicle suspension

In essence, a mechanical-based inerter is a physical device which achieves the mass-like effect by exploiting the rotational inertia and converting it to two-terminal, translational inertia effect by means of motion conversion mechanisms, such as ball-screw mechanism [17], rack-and-pinion mechanism [17], hydraulic mechanism [18], etc. In a more recent realization, the inertial effect of moving fluid in helical tube has also been adopted [19,20], which allows the inerter to be controllable [20]. Another recently studied realization uses the crank mechanism to achieve the intended inertial effect and also to provide a variable negative stiffness [21]. As a passive element, working principle of an inerter follows the definition as in Eq (1), as first stated in [2]:

$$F_{inerter}=b\left(a_{2}-a_{1}\right)$$ (1)

in which *F*_*inerter*_ is the force at the terminals, *b* is the property of the inerter known as the inertance with the unit of kilogram (kg), while *a*_1_ and *a*_2_ are the relative acceleration of the terminals. In other words, an inerter has the property that the force exerted is directly proportional to the relative acceleration at its terminals. From a microscopic point of view, each of the physical realizations of inerter device described earlier has its own detailed derivation in which the inertance is a function of its design variables, and each also has some non-ideal factors apart from the inertial effect, such as inherent elasticity, friction, and backlash as reported previously [22], and inherent damping as well for the fluid inerter [19]. However, macroscopically, all these still follows the ideal force-acceleration relationship, very much like the displacement-responding behavior of a suspension spring and the velocity-responding behavior of a suspension damper. This is the fundamental inerter definition that is employed in this study, following most of the previous studies concerning inerter in vehicle suspensions [10–14].

**Fig 1:**
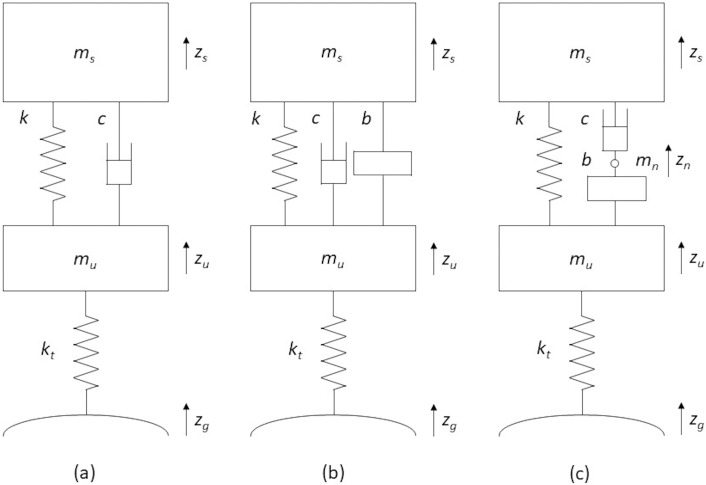
A quarter vehicle model with (a) typical spring and damper suspension, (b) parallel inerter suspension layout, and (c) serial inerter suspension layout.

In the context of vehicle suspension, there can be many layouts of suspension components that the addition of an inerter can form, many of which can be seen in [10] and [11]. However, presently, the most common suspension layouts with inerter remain the simple parallel and the simple serial inerter layouts. As the name implies, the former has the inerter added in parallel to the spring and damper elements to form a suspension, while the latter contains a serially arranged inerter to the usual vehicle suspension with spring and damper. Within the same context of study, these layouts are often implemented in a two-degree-of-freedom (DOF) quarter vehicle model for theoretical performance analysis and optimization [10–16]. Following this, these layouts, together with the quarter vehicle model, are illustrated in Fig 1.

In which *m*_*s*_, *m*_*u*_ are the sprung and unsprung masses, *k* is the suspension stiffness, *c* is the suspension damping, *b* is the inertance of the added inerter, *k*_*t*_ is the tire stiffness, and *z*_*s*_, *z*_*u*_, *z*_*g*_ are vertical sprung mass displacement, unsprung mass displacement and the road input displacement respectively. For serial inerter, *m*_*n*_ represents the mass of the node that connects between the damper and the inerter, while *z*_*n*_ is the corresponding displacement.

The additional inerter component in Fig 1b and 1c expectedly changes the equations of motion of a quarter vehicle model, albeit slightly. Therefore, when employed in studies involving vehicle suspension, the equation sets as below are applicable. For parallel inerter layout, the dynamics of motion are detailed by Eqs (2) and (3):

$$m_{s}{\ddot{z}}_{s}=k\left(z_{u}-z_{s}\right)+c\left({\dot{z}}_{u}-{\dot{z}}_{s}\right)+b\left({\ddot{z}}_{u}-{\ddot{z}}_{s}\right)$$ (2)

$$m_{u}{\ddot{z}}_{u}=k_{t}\left(z_{g}-z_{u}\right)-k\left(z_{u}-z_{s}\right)-c\left({\dot{z}}_{u}-{\dot{z}}_{s}\right)-b\left({\ddot{z}}_{u}-{\ddot{z}}_{s}\right)$$ (3)

Meanwhile, for serial inerter layout, Eqs (4) to (6) are applicable:

$$m_{s}{\ddot{z}}_{s}=k\left(z_{u}-z_{s}\right)+c\left({\dot{z}}_{n}-{\dot{z}}_{s}\right)$$ (4)

$$m_{n}{\ddot{z}}_{n}=b\left({\ddot{z}}_{u}-{\ddot{z}}_{n}\right)-c\left({\dot{z}}_{n}-{\dot{z}}_{s}\right)$$ (5)

$$m_{u}{\ddot{z}}_{u}=k_{t}\left(z_{g}-z_{u}\right)-k\left(z_{u}-z_{s}\right)-b\left({\ddot{z}}_{u}-{\ddot{z}}_{n}\right)$$ (6)

The aforementioned combinations of vehicle model and suspension layout are the common setup as seen in various studies concerning inerter in vehicle suspension. In all these studies, suspension performance improvements have been shown. For instance, in the early work of [10] and [11], the optimization and analytical solution involving vehicle models with various suspension layouts involving inerter gave improvements in ride comfort, tire load and suspension’s ability to carry load, for a wide range of suspension stiffness. Similarly, in another study [12], the parallel inerter in a quarter vehicle model was subjected to alternative goal programming optimization method, and superior passenger comfort and tire grip were obtained while maintaining equal suspension deflection compared to conventional passive suspension without inerter. Later, with the implementation of variable or switchable inerter in mathematical vehicle models, further enhancement in suspension performance has also become achievable [14]. In a recent study, an inerter-based mechatronic device, consisting of an inerter and an electric motor with passive load, was used to achieve vehicle vibration suppression, and the study demonstrated improvements in road holding without diverse effect on ride comfort and suspension travel [15]. Meanwhile, another study showed the inerter being implemented with an active suspension to improve ride comfort and reduce actuator force of the active suspension [16].

While the above, among others, all lead to the consistent point that the inerter is capable of giving superior performance when adopted in the suspension of passenger cars, presently there has not been similar performance study that is specifically meant for heavy vehicles. As mentioned earlier, the implementation of inerter is still new in heavy vehicles. Since heavy vehicles differ vastly from passenger cars in terms of vehicle parameter values, it is worth to consider a few heavy vehicle types and investigate the suspension performance due to the implementation of inerter in these vehicles.

## Vehicle modeling and setup of analysis

In line with earlier studies of inerter, the two-DOF quarter vehicle model was taken as the representative system of vehicle in this study. This is a lumped-mass, rigid body mathematical model that is commonly used in studies concerning fundamental suspension analyses, including inerter and semi-active suspensions [14]. While maintaining simplicity, it can be used to obtain qualitatively correct sprung and unsprung mass responses [23] for comparative analyses, and adequate accuracy are achievable without resorting to higher-DOF models [24]. For comprehensiveness, the quarter vehicle parameter values of a truck and a bus were adopted, as both these heavy vehicle classes were of interest in the study of inerter’s performance benefit. These are presented in Table 1 together with those of a typical passenger car as reference [14,25,26]. By quick observation, it is worth noting that each parameter value of heavy vehicles is generally an order of magnitude greater than the corresponding parameter value of a passenger car.

**Table 1:** The quarter vehicle parameter values of a truck and a bus with a typical passenger car as comparison.

Vehicle parameter	Truck	Bus	Passenger car
Sprung mass, *m*_*s*_ (kg)	3400	4000	317.5
Unsprung mass, *m*_*u*_ (kg)	350	550	45.4
Suspension stiffness, *k* (Nm^-1^)	300000	200000	22000
Suspension damping, *c* (Nsm^-1^)	2000 / 20000 (compression / expansion)	10000	1500
Tire stiffness, *k*_*t*_ (Nm^-1^)	1000000	1700000	192000

**Fig 2:**
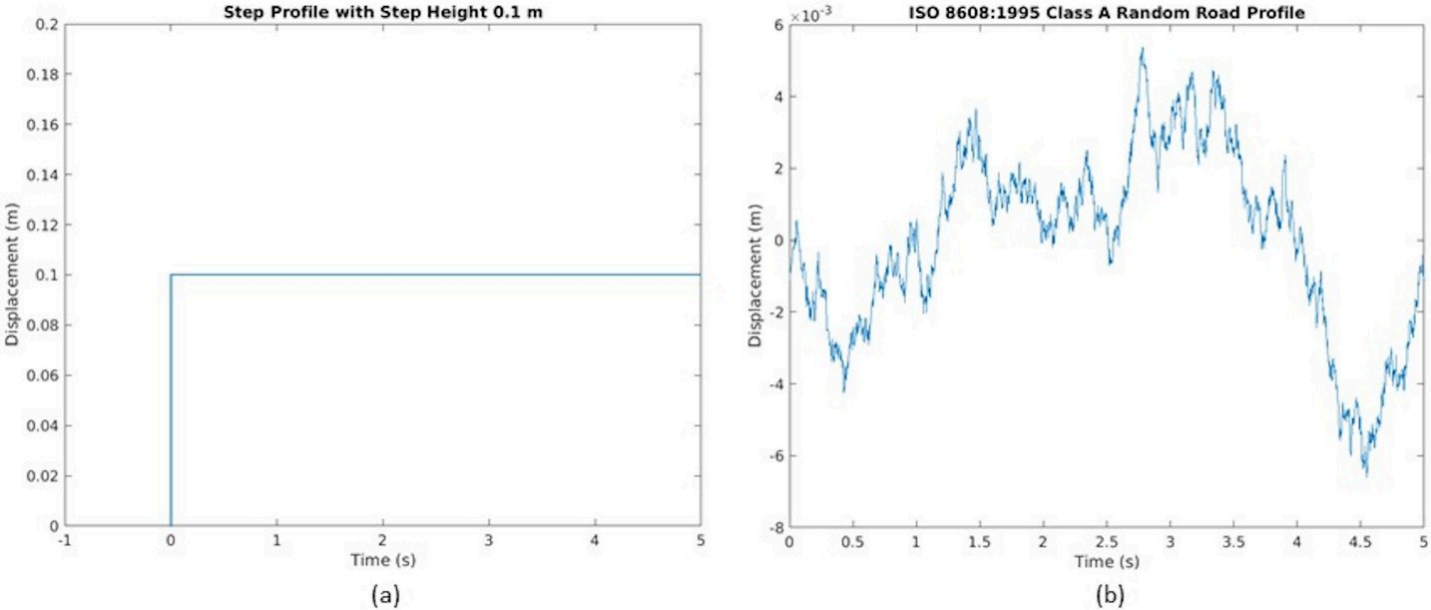
Representation of (a) step road profile of 0.1 m height and (b) random smooth road profile in the study.

The two vehicle models were constructed in computational software environment (Simulink^®^), following the dynamics of parallel inerter layout (Eqs (2) and (3)) and serial inerter layout (Eqs (4) to (6)). Combined with the road profile modeling, the models can be simulated to obtain the sprung and unsprung mass responses due to the presence of a vertical road displacement input. As shown in Fig 2, two different profiles were chosen as the road input, namely the step road profile and the random road profile. The step road profile is a transient input with a fixed step height that is also common in control system studies; it was adopted for a simplified emulation of a vehicle hitting an obstacle, such as a curb or a bump. Meanwhile, the random road profile is a realistically-generated profile based on the road roughness coefficient from ISO8608:1995 (class A; smooth road classification) [27]; it was used in this study to emulate regular smooth-road driving, for example as seen in long-distance expressway transportation involving heavy vehicles. The use of both road inputs in the study ensures comprehensive coverage of driving scenarios, since a road is regarded as a combination of isolated transient road features and continuously distributed profile irregularities [23].

The suspension performance improvements due to inerter were investigated via two separate approaches. In the first approach, the inerter was treated solely as an add-on suspension component to the existing quarter vehicle models to emulate retrofitting a suspension, thus having the suspension stiffness and damping maintained. This is applicable to both analyses involving the parallel inerter layout and the serial inerter layout. Meanwhile, in the second approach, both the inertance and the damping rate were subjected to optimization of suspension performance criteria to determine the performance benefit. This emulates suspension tuning from scratch. It is also in accordance to past research which suggests combined consideration of inerter and damping in vehicle suspension implementation [13]. For both approaches mentioned above, a range of *b* from 0 kg to 1000 kg was tested for the parallel inerter layout, while the range of 0 kg to 3000 kg was applicable to the serial inerter layout. These are in line with similar past studies which considered inertance to sprung mass ratio of close to one as the evaluation limit [12–14]. Meanwhile, the parallel inerter layout was assessed with a smaller range due to small values of optimum inertance reported in past studies involving passenger cars. These ranges remain generally realistic as large inertance values are achievable by appropriate design values of the design function *b* while keeping reasonable actual device mass [2]. Meanwhile, the damping took a range from 0 Nsm^-1^ to 30000 Nsm^-1^ in the optimization of second approach of study. In general, all these ranges are wider than those adopted in similar past studies involving passenger cars, since the heavy vehicle parameter values are greater, and by consistent scaling considering transfer function, the suitable inertance (and damping if optimized together) is expectedly greater as well.

## Suspension performance with inerter

From the computational results corresponding to the two quarter vehicle models with inerter, the performance of heavy vehicle suspension with inerter was looked into, and any benefit of incorporating inerter in the suspension of heavy vehicles would be known. In the study, two suspension performance criteria were considered, namely the root-mean-squared (RMS) sprung mass acceleration and the RMS dynamic tire load. Basically, the former describes the effect on the sprung mass (vehicle body) due to road input and is a measure of vehicle ride comfort, while the latter represents the variation of tire loads on the ground, which affects the tire-ground contact and therefore is a measure of road holding ability. In both considerations of implementation (inerter as add-on component and inerter with damping considered), reductions of RMS sprung mass acceleration and RMS dynamic tire load represent respectively the improvements in ride and road holding of the tested vehicle models.

### Inerter as add-on component

**Fig 3:**
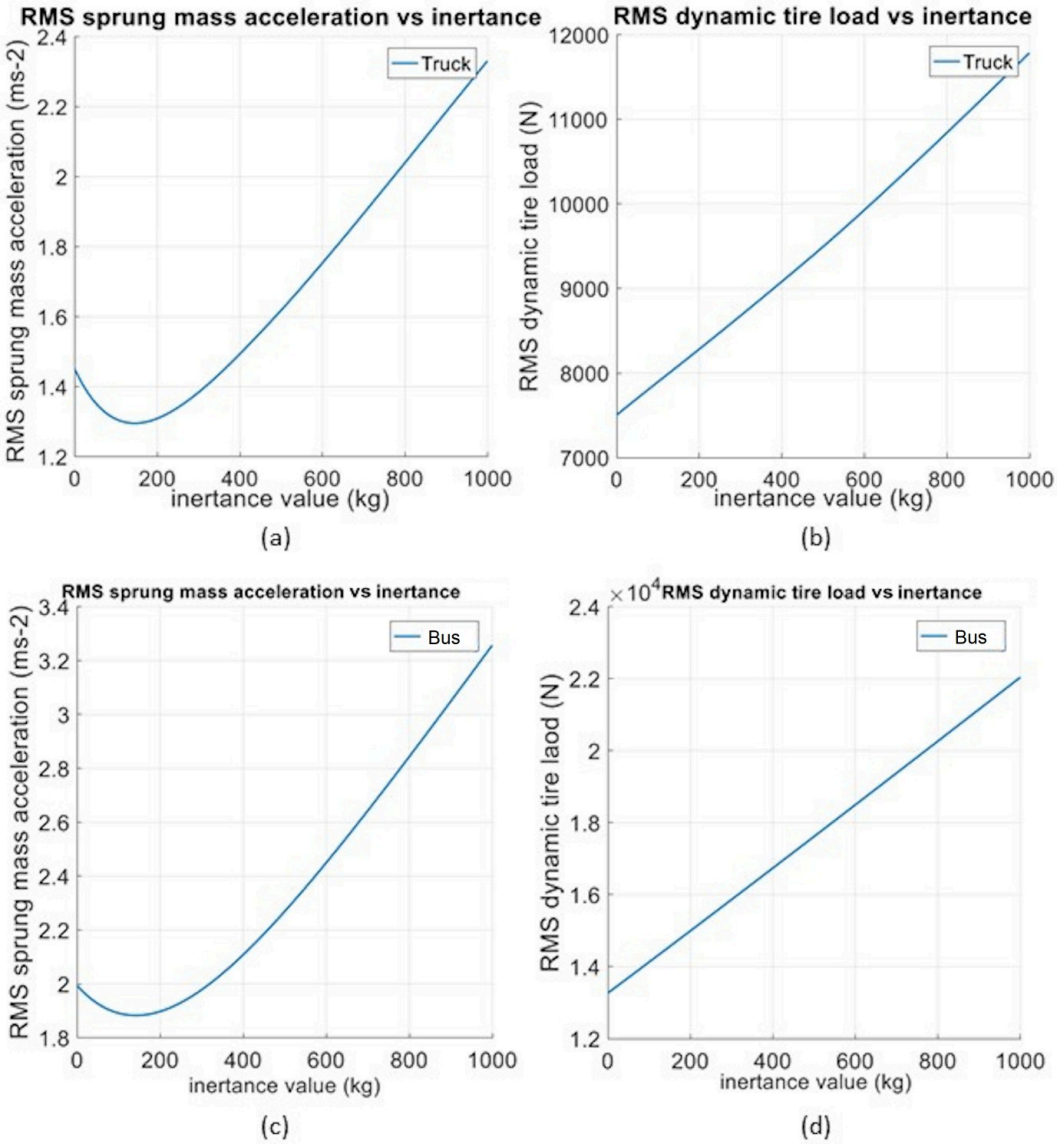
(a) RMS sprung mass acceleration and (b) RMS dynamic tire load against parallel inertance for truck, with (c) RMS sprung mass acceleration and (d) RMS dynamic tire load variation for bus.

Fig 3 displays the RMS sprung mass acceleration and RMS dynamic tire load with parallel inertance value *b* for the truck and bus models, respectively, under the step road input of 0.1 m. The responses of truck and bus models are of similar trend, for which the RMS sprung mass acceleration shows reduction while the RMS dynamic tire load increases with *b*. It can be observed from Fig 3a and 3c that there are absolute minimum RMS sprung mass accelerations of 1.296 ms^-2^ and 1.883 ms^-2^ when the *b* values are 145 kg and 140 kg, respectively, for the truck and bus models. Meanwhile, there is no minimum RMS dynamic tire load observed for both models. In fact, at the ride-optimized *b* values, the RMS dynamic tire loads for the truck and bus models are greater at 8.070 kN and 14.474 kN, respectively, as displayed in Fig 3b and 3d.

In the context of suspension performance, the add-on parallel inerter with step road input of 0.1 m has resulted in reductions of 10.67% and 5.54% in RMS sprung mass acceleration, respectively, for the truck and bus models. These are, in fact, greater improvements compared to that achieved for some tested passenger car in earlier studies (vehicle parameter values as stated in Table 1; about 2%) [13–14]. Therefore, it is worth noting that the implementation of add-on parallel inerter is also effective on improving the ride performance of heavy vehicles in addition to passenger cars. It is also interesting to note that both the truck and bus models have optimum *b* values (145 kg and 140 kg) that are two orders of magnitude greater compared to that of a typical passenger car [13]. This can be attributed to the consistent scaling-up of vehicle parameter values for heavy vehicles relative to passenger cars, which is apparent in Table 1. Meanwhile, in term of RMS dynamic tire load, the add-on parallel inerter has caused increments of 7.53% and 8.92%, respectively, for the truck and bus models. The increments are slightly greater than that in the relevant earlier studies concerning a passenger car (about 5%), but generally the level of increment in RMS dynamic tire load is almost the same, hence the road holding ability is relatively maintained.

Table 2 summarizes the suspension performance of truck and bus models with add-on parallel and serial inerters under different road inputs. For the add-on parallel inerter with random road input, both the truck and bus models perform similarly as in the step road input situation, where there is an improvement of ride comfort at the expense of road holding ability. For RMS sprung mass acceleration, the truck and bus models display reductions of 4.19% and 6.20%, respectively. Meanwhile, there are increments of RMS dynamic tire load of 7.11% and 9.88% for the truck and bus models, respectively.

**Table 2:** Summary and comparison of heavy vehicle suspension performance due to different road inputs.

		RMS sprung mass acceleration (ms^-2^)			RMS dynamic tire load (kN)		
**Truck**		**Reference**	**Optimum**	**Difference (%)**	**Reference**	**Optimum**	**Difference (%)**
Step road input	Parallel	1.4504	1.2957	-10.67	7.5045	8.0698	7.53
	Serial	1.4504	2.9030	100.15	7.5045	12.0476	60.54
Random road input	Parallel	0.1767	0.1693	-4.19	0.7695	0.8242	7.11
	Serial	0.1767	0.1996	12.96	0.7695	0.9562	24.26
**Bus**		**Reference**	**Optimum**	**Difference (%)**	**Reference**	**Optimum**	**Difference (%)**
Step road input	Parallel	1.9933	1.8828	-5.54	13.2644	14.4474	8.92
	Serial	1.9933	2.4342	22.12	13.2644	15.0270	13.29
Random road input	Parallel	0.1773	0.1663	-6.20	1.2573	1.3815	9.88
	Serial	0.1773	0.1761	-0.68	1.2573	1.3220	5.15

In comparison of the effect of different road inputs, it can be observed that the add-on parallel inerter (truck model) achieves greater reduction in RMS sprung mass acceleration with step road input compared to random road input. This implies that the ride comfort improvement of the truck model is greater in transient scenarios such as road bumps. Conversely, the ride improvements for the bus model, even though exist for the add-on parallel inerter, are about the same between the two road inputs. From these comparisons, the performance benefits due to the incorporation of inerter on vehicle suspensions are generally affected by the combinations of vehicle parameter values that differ for different vehicle categories.

With step road profile as the road input, it is also possible to investigate the ride performance from the perspective of transient response characteristics. In particular, reasonably long rise time and peak time, as well as low percentage overshoot and short settling time, are some of the characteristics of a vehicle with good ride comfort. Table 3 displays the transient response characteristics of the truck and bus models with parallel and serial inerter layouts. The results show that the rise time and peak time can be increased by 8.47% and 2.39%, respectively, while the percentage overshoot and settling time can be reduced by 10.32% and 15.90%, respectively, for the truck model with add-on parallel inerter. These indicate reasonably good ride comfort improvement. Meanwhile, the bus model with add-on parallel inerter shows 5.88% increment in rise time, but just a slight increase in peak time. Additionally, the reduction in percentage overshoot of 2.47% is less than that of the truck model, while the settling time is increased by 1.19%. Consistent with the earlier deduction that the performance benefits are dependent on vehicle categories, it is quite obvious here that the truck model has better ride improvement compared to the bus model.

**Table 3:** Transient response characteristics of heavy vehicles with inerter due to step road input.

	Reference	Optimum (parallel)	Difference (%)	Optimum (serial)	Difference (%)
**Truck**					
Rise time (s)	0.177	0.192	8.47	0.149	-15.82
Peak time (s)	0.419	0.429	2.39	0.420	0.24
Maximum overshoot (%)	43.568	39.071	-10.32	92.589	112.52
Settling time (s)	3.888	3.270	-15.90	5.000	28.60
**Bus**					
Rise time (s)	0.119	0.126	5.88	0.129	8.40
Peak time (s)	0.359	0.360	0.28	0.389	8.25
Maximum overshoot (%)	0.748	0.730	-2.47	0.836	11.68
Settling time (s)	4.281	4.332	1.19	5.000	16.80

Conversely, the implementation of serial inerter as add-on component to the suspension of heavy vehicle is much less effective relative to parallel inerter at the existing damping rate. There is basically no absolute minimum for RMS sprung mass acceleration and RMS dynamic tire load across the range of inertance values *b*. Consequently, in order to compare with the add-on parallel inerter, the *b* values at which changes in both RMS values become small and insignificant were chosen, namely 800 kg and 1000 kg for the truck and bus models, respectively. The results show that the add-on serial inerter does not improve the responses due to road inputs, for both the truck and bus models. From Table 2, increments in RMS sprung mass acceleration and RMS dynamic tire load are observed, except for a very slight and rather negligible reduction in RMS sprung mass acceleration corresponding to the random road input. There is also little improvement in the transient response, as evident from Table 3.

Even though the serial inerter layout does not bring performance benefits when considered as an add-on component to existing suspensions, it is important not to rule it out for further investigation. Due to the sequential arrangement between suspension damper and inerter, it is possible that the add-on approach to existing damping rate is not exactly optimum yet for the serial inerter layout. In fact, it has been suggested before that the suspension damping *c* should be adjusted together with inertance *b* to harness more potential from inerter [13]. Thus, it will be good if both *b* and *c* values are considered in the optimization for ride and road holding ability. The results are discussed in the next sub-section.

### Optimized performance based on inertance and damping

**Fig 4:**
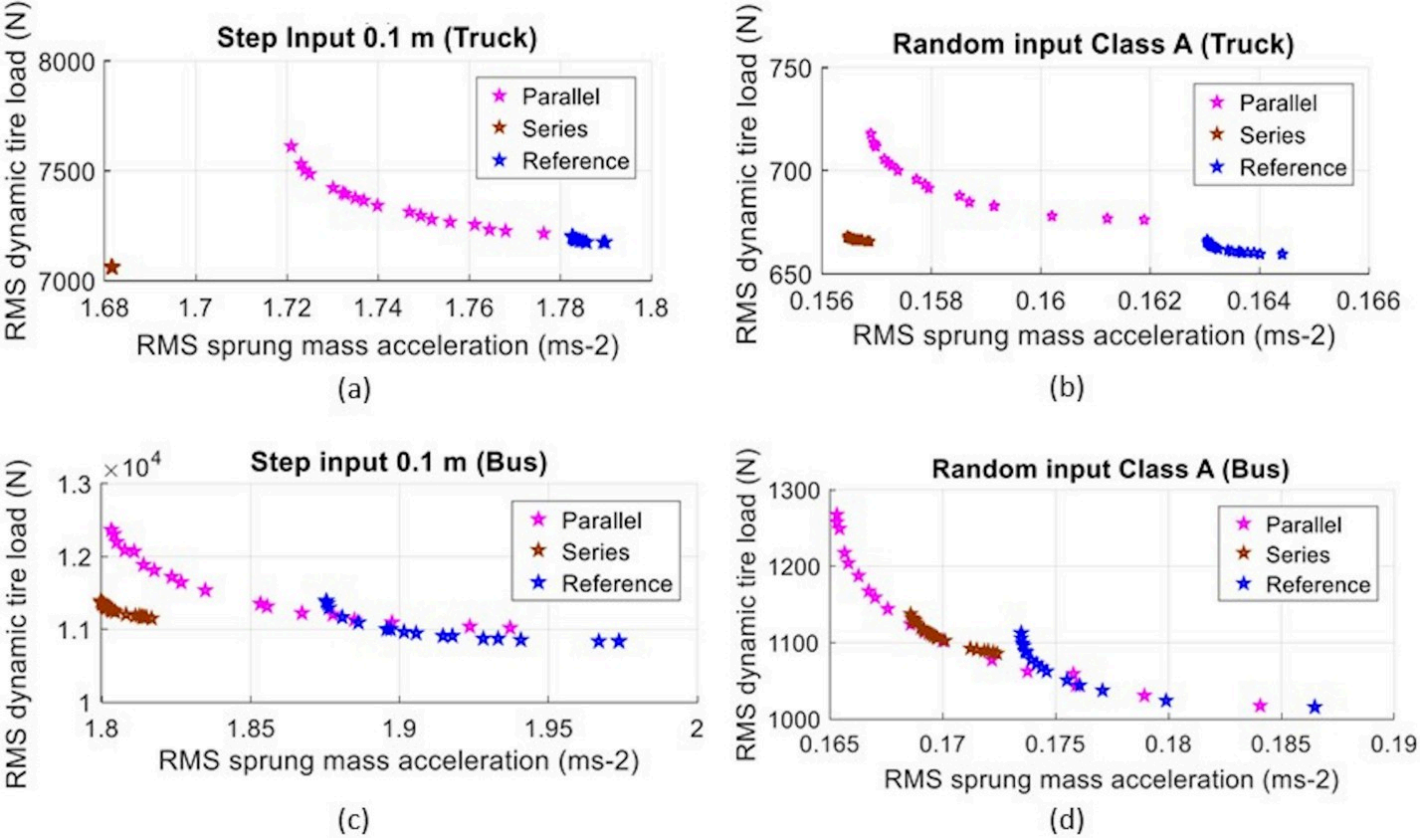
Pareto fronts for truck considering (a) step road input and (b) random road input, with the same optimization for bus considering (c) step road input and (d) random road input.

In the last part of the study, both inertance *b* and suspension damping *c* were optimized based on the same suspension performance criteria, namely the RMS sprung mass acceleration, an indication of ride comfort, and the RMS dynamic tire load, an indication of road holding ability. However, both criteria were analyzed together in the optimization. Admittedly, the nature of ride comfort and road holding ability are known to be conflicting among each other. Generally, an emphasis towards the former will lead to a compromise of the latter. Therefore, to optimize, or minimize, both RMS sprung mass acceleration and RMS dynamic tire load, the Pareto optimization approach considering these two as optimization objectives were adopted. The idea of Pareto optimization is that the solutions obtained are non-dominated solutions, as none of these can minimize one objective without worsening another. It follows that the set of optimum solutions are equally dominant with only different emphasis on each objective. Using the specified ranges of inertance *b* and damping *c* as described in the vehicle modeling section, the optimum solutions considering the two objectives are shown as Pareto fronts for the truck and bus models with parallel and serial layouts of inerter. These are illustrated in Fig 4. They are also compared with the respective optimum solutions for the reference case without inerter.

In a qualitative comparison, a case is superior to another if its Pareto front is towards the direction of minimizing the objectives, relative to that of another. Fig 4a and 4b show that the serial inerter layout for the truck model displays quite promising results, as it reduces the RMS sprung mass acceleration without worsening the RMS dynamic tire load. Meanwhile, the parallel inerter layout shows a shift of Pareto front towards the left but upwards when compared to the reference. This infers that it is possible to reduce the RMS sprung mass acceleration but with a corresponding increase in RMS dynamic tire load, which is quite similar to the performance of add-on parallel inerter in the previous sub-section. As an overall observation, when both inertance *b* and damping *c* are allowed to vary, the suspension with serial inerter is superior to the suspension with parallel inerter which, in turn, is better than the reference suspension without inerter (the spring-and-damper configuration). This is applicable to step road input and random road input. Additionally, Pareto fronts for the bus model also display the same trend as that for the truck model. In general, the implementation of inerter in the suspension of heavy vehicles does bring better ride comfort while maintaining the road holding ability.

Finally, to evaluate the suspension performance quantitatively, the middle point of the Pareto front from each case is selected as the sample solution for comparison, as shown in Table 4. Each point on the Pareto front corresponds to a set of RMS values which can be mapped back to some combination of *b* and *c* values. The approach of taking the middle point from each set of solutions brings a balanced emphasis on the minimization of both objectives. This allows a fair comparison of performance improvements among different layouts of inerter, as well as different types of heavy vehicle.

**Table 4:** Summary and comparison of heavy vehicle suspension performance considering optimization of damping and inertance.

		RMS sprung mass acceleration (ms^-2^)			RMS dynamic tire load (kN)		
**Truck**		**Reference**	**Optimum**	**Difference (%)**	**Reference**	**Optimum**	**Difference (%)**
Step road input	Parallel	1.784	1.740	-2.45	7.1843	7.3414	2.19
	Serial	1.784	1.682	-5.72	7.1843	7.0627	-1.69
Random road input	Parallel	0.163	0.158	-3.22	0.6624	0.6917	4.42
	Serial	0.163	0.157	-4.05	0.6624	0.6665	0.62
**Bus**		**Reference**	**Optimum**	**Difference (%)**	**Reference**	**Optimum**	**Difference (%)**
Step road input	Parallel	1.896	1.835	-3.21	11.0058	11.5281	4.75
	Serial	1.896	1.808	-4.62	11.0058	11.2114	1.87
Random road input	Parallel	0.176	0.170	-3.15	1.0500	1.1030	5.04
	Serial	0.176	0.172	-2.01	1.0500	1.0885	3.66

The truck model with serial inerter displays the greatest reductions in the RMS sprung mass acceleration, with 5.72% and 4.05% observed for the step and random road inputs, respectively, without significant changes in the RMS dynamic tire load. In fact, the RMS dynamic tire load is actually also reduced by 1.69% with the case involving step road input. Meanwhile, the truck model with parallel inerter shows 2.45% and 3.22% reductions in the RMS sprung mass acceleration, with 2.19% and 4.42% increments in RMS dynamic tire load, respectively, for the step and random road inputs. Thus, the serial inerter layout does indeed outperform the parallel inerter layout for the truck model, as the former is capable of achieving better ride comfort and road holding ability. It is now apparent that when the suspension damping is tuned together with the inertance, the benefit brought by a serial inerter can be boosted further.

For the bus model, the serial inerter gives slightly more reduction of 4.62% in the RMS sprung mass acceleration with the step road input, while showing less reduction with the random road input (2.01%). The indication that the inerter gives better transient response, as first pointed out in the previous sub-section, is again observed here. Meanwhile, the reduction of RMS sprung mass acceleration for the parallel inerter is similar between the two road input cases (approximately 3.2% reduction is achievable). Comparing by using the bus model, the serial inerter layout is again superior to the parallel inerter layout as the former has less increment of the RMS dynamic tire load, hence less compromise to the road holding ability when the ride is improved.

## Conclusion

This study investigates the inerter-based suspension systems for heavy vehicles, and it shows that the inerter brings superior performance compared to a suspension without inerter. Results have shown that the inerter is capable of improving two major suspension performance criteria, whether it is implemented in the parallel or serial layout. When the inerter is treated as an add-on device, the parallel inerter layout improves the sprung mass acceleration at the expense of dynamic tire load. Meanwhile, when both inertance and suspension damping are considered, the serial inerter demonstrated superiority with improvements in both sprung mass acceleration and dynamic tire load, although this comes with narrower Pareto optimal design points than that of the parallel inerter layout. Regardless of layouts, the level of performance improvement for heavy vehicles is comparable to that for passenger cars with improvements of up to 10%, but generally the required optimum inertance for heavy vehicles is two orders of magnitude greater than that for passenger cars. Overall, this study has demonstrated that the inerter has similar improvements in vibration isolation and road holding performance when incorporated in heavy vehicles instead of in passenger cars where the improvements are already known. The benefits that these translate to, namely better passenger comfort and prevention of goods damage, together with the greater installation space and less critical penalty of weight due to the installation of an additional device, make the mass-adoption of inerter in heavy vehicles especially promising.

## Supporting information

S1 File Structure of quarter vehicle model with parallel inerter in Simulink^®^. (PDF)

S2 File Structure of quarter vehicle model with serial inerter in Simulink^®^. (PDF)
