# Correction: Investigation of inerter-based suspension systems for heavy vehicles

**DOI:** 10.1371/journal.pone.0286178

**Published:** 2023-05-18

**Authors:** Ming Foong Soong, Rahizar Ramli, Ahmad Abdullah Saifizul, Kah Yin Goh, Su Xian Long

There are errors in the Funding statement. The correct Funding statement is as follows: This work is supported by Fundamental Research Grant Scheme (Grant number: FRGS/1/2020/TK0/UM/02/23, Institution project number: FP086-2020), Ministry of Higher Education of Malaysia (Recipient: Saifizul, A. A.), and Faculty Research Grant Scheme (GPF018A-2018), Universiti Malaya (Recipient: Ramli, R.). The funders had no role in study design, data collection and analysis, decision to publish, or preparation of the manuscript.
