# Supplementary material for: Investigation of inerter-based suspension systems for heavy vehicles
Source: PLoS One. 2023 Jan 20;18(1):e0280290. doi: 10.1371/journal.pone.0280290 (PMC9858774; doi:10.1371/journal.pone.0280290)
Supplement: S1 File — (PDF) [file pone.0280290.s001.pdf]

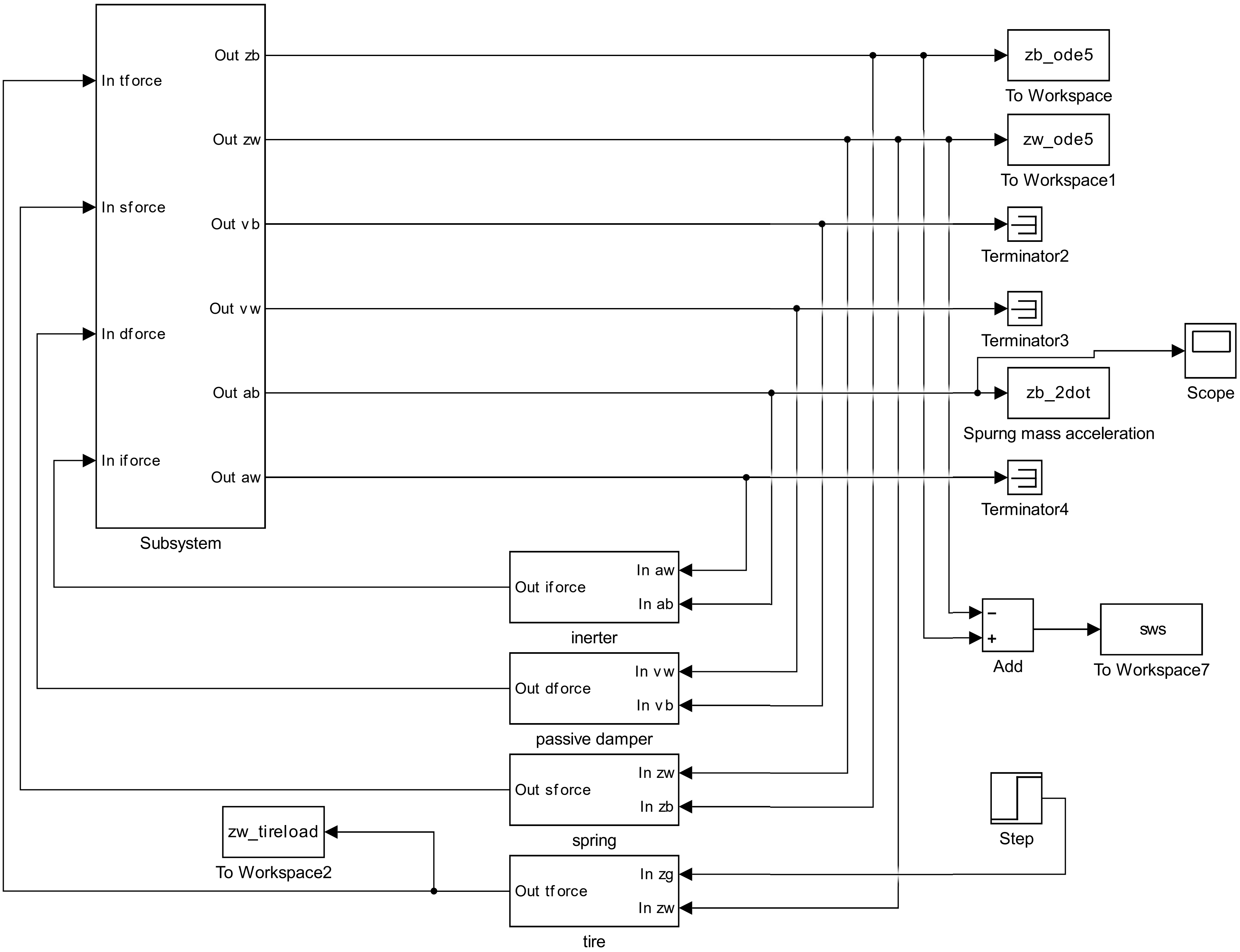

In tforce
In sforce
In dforce
In iforce
Out zb
Out zw
Out vb
Out vw
Out ab
Out aw
Subsystem
zb_ode5
To Workspace
zw_ode5
To Workspace1
Terminator2
Terminator3
Scope
zb_2dot
Spurng mass acceleration
Terminator4
In aw
Out iforce
In ab
inerter
In vw
Out dforce
In vb
passive damper
In zw
Out sforce
In zb
spring
zw_tireload
To Workspace2
In zg
Out tforce
In zw
tire
Add
sws
To Workspace7
Step

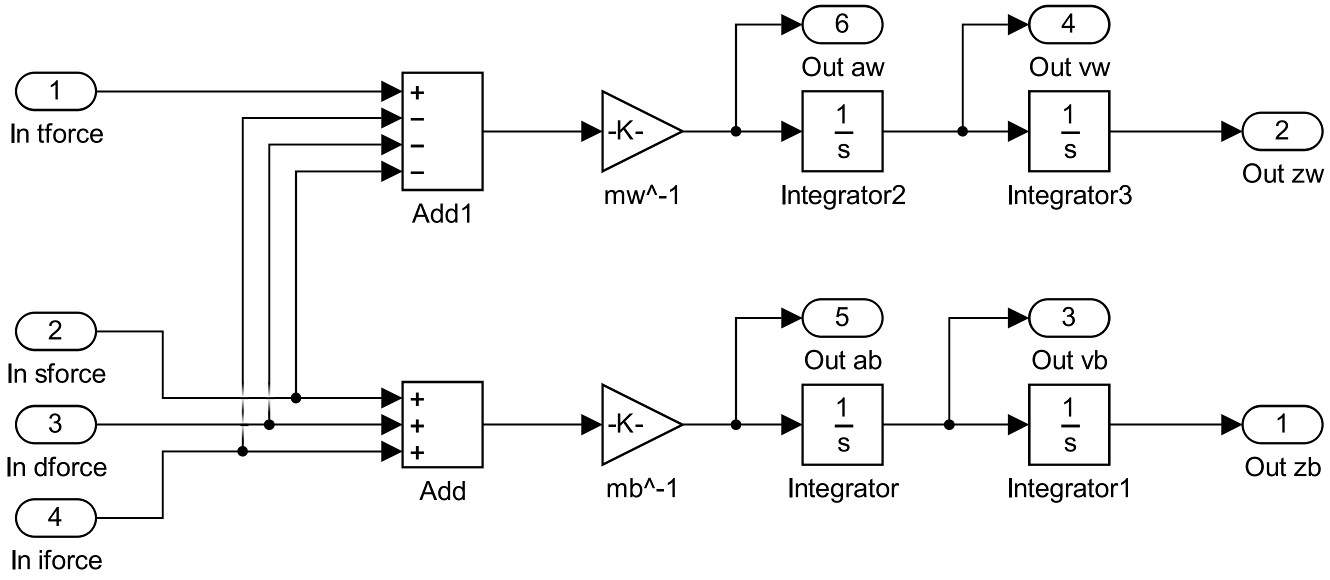
1
In tforce
2
In sforce
3
In dforce
4
In iforce
+
−
−
−
Add1
+
+
+
Add
-K-
mw^-1
-K-
mb^-1
6
Out aw
1/s
Integrator2
4
Out vw
1/s
Integrator3
2
Out zw
5
Out ab
1/s
Integrator
3
Out vb
1/s
Integrator1
1
Out zb

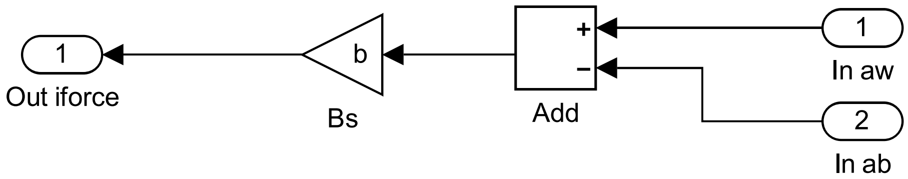
1
Out iforce
b
Bs
+
−
Add
1
In aw
2
In ab

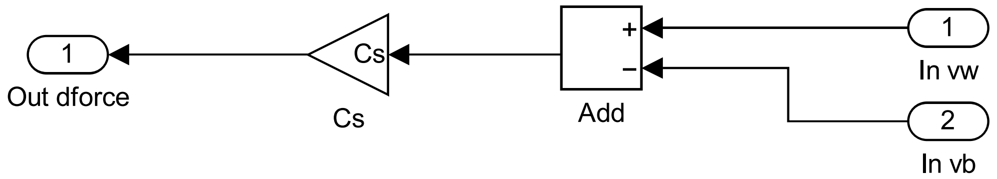
1
Out dforce
Cs
Cs
+
−
Add
1
In vw
2
In vb

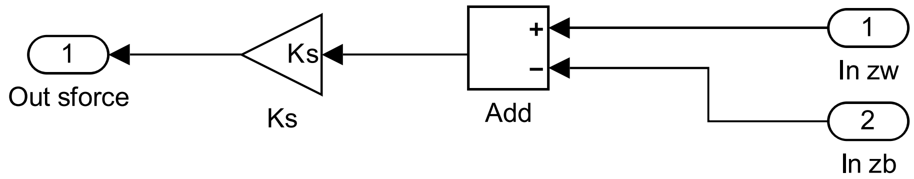
1
Out sforce
Ks
Ks
+
−
Add
1
In zw
2
In zb

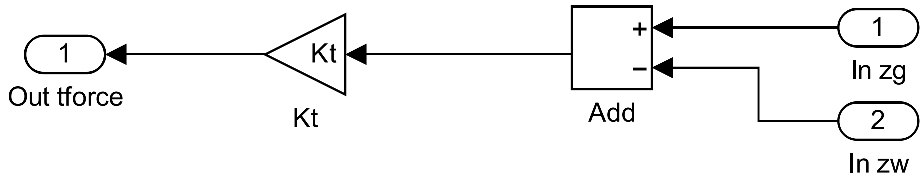
1
Out tforce
Kt
Kt
+
−
Add
1
In zg
2
In zw

Page	System Name
1	two_dof_passive_step_1
2	two_dof_passive_step_1/Subsystem
3	two_dof_passive_step_1/inerter
4	two_dof_passive_step_1/passive damper
5	two_dof_passive_step_1/spring
6	two_dof_passive_step_1/tire
