# Supplementary material for: Investigation of inerter-based suspension systems for heavy vehicles
Source: PLoS One. 2023 Jan 20;18(1):e0280290. doi: 10.1371/journal.pone.0280290 (PMC9858774; doi:10.1371/journal.pone.0280290)
Supplement: S2 File — (PDF) [file pone.0280290.s002.pdf]

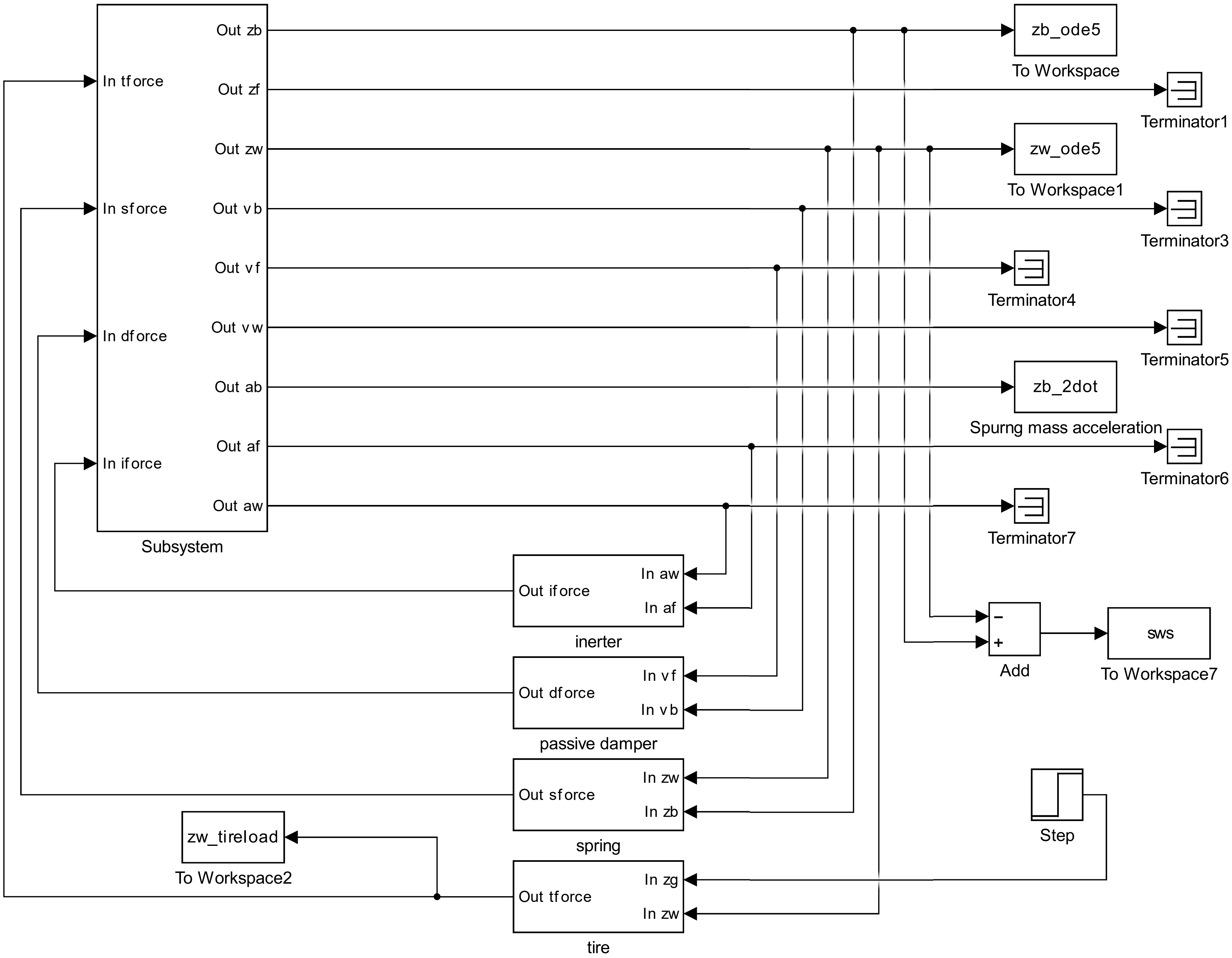

Out zb
In tforce
Out zf
Out zw
In sforce
Out vb
Out vf
In dforce
Out vw
Out ab
Out af
In iforce
Out aw
Subsystem
zb_ode5
To Workspace
Terminator1
zw_ode5
To Workspace1
Terminator3
Terminator4
Terminator5
zb_2dot
Spurng mass acceleration
Terminator6
Terminator7
In aw
Out iforce
In af
inerter
In vf
Out dforce
In vb
passive damper
In zw
Out sforce
In zb
spring
zw_tireload
To Workspace2
In zg
Out tforce
In zw
tire
Add
sws
To Workspace7
Step

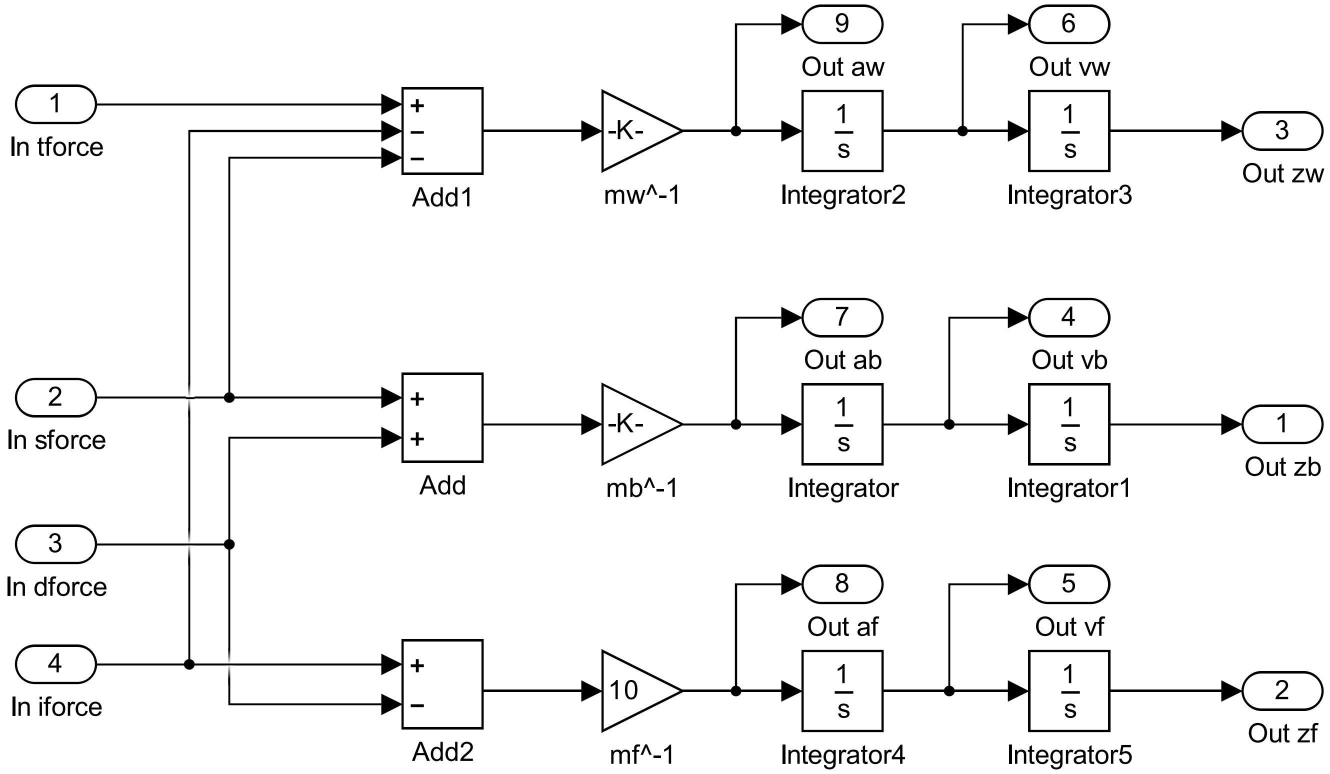

1
In tforce
2
In sforce
3
In dforce
4
In iforce
+
−
−
Add1
+
+
Add
+
−
Add2
-K-
mw^-1
-K-
mb^-1
10
mf^-1
9
Out aw
6
Out vw
7
Out ab
4
Out vb
8
Out af
5
Out vf
1/s
Integrator2
1/s
Integrator3
1/s
Integrator
1/s
Integrator1
1/s
Integrator4
1/s
Integrator5
3
Out zw
1
Out zb
2
Out zf

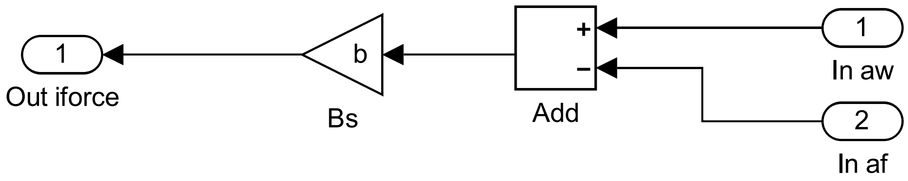
1
Out iforce
b
Bs
+
−
Add
1
In aw
2
In af

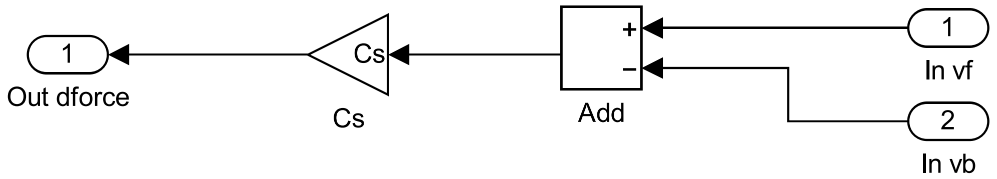
1
Out dforce
Cs
Cs
Add
+
−
1
In vf
2
In vb

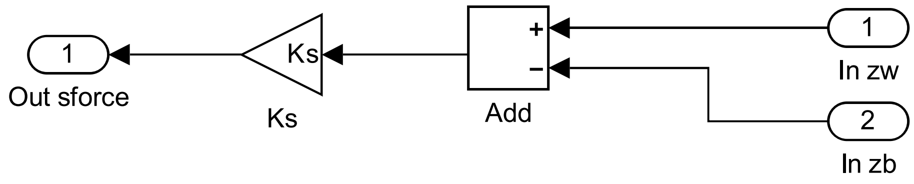
1
Out sforce
Ks
Ks
+
−
Add
1
In zw
2
In zb

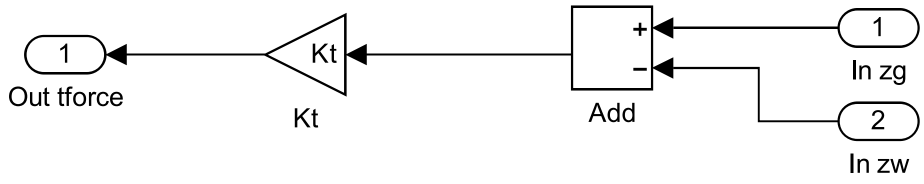
1
Out tforce
Kt
Kt
+
−
Add
1
In zg
2
In zw

Page	System Name
1	two_dof_passive_step_2
2	two_dof_passive_step_2/Subsystem
3	two_dof_passive_step_2/inerter
4	two_dof_passive_step_2/passive damper
5	two_dof_passive_step_2/spring
6	two_dof_passive_step_2/tire
